# A 3-D Metal-Organic Framework Constructed with Manganese(ΙΙ), 4,4'-Oxybis(benzoic acid) and 2,2'-Biphenyl: Synthesis, Crystal Structure and Photoelectric Property Characterization

**DOI:** 10.3390/molecules170911103

**Published:** 2012-09-14

**Authors:** Kai-Sheng Diao, Long Li, Yu-Qiu Ding, Fu-Hou Lei

**Affiliations:** 1 College of Chemistry and Chemical Engineering, Guangxi University for Nationalities, Nanning 530006, China; Email: diaokaisheng@126.com (K.-S.D.); houfanlilong@126.com (L.L.); daaiwujiang@163.com (Y.-Q.D.); 2 Guangxi Key Laboratory of Chemistry and Engineering of Forest Products, Guangxi University for Nationalities, Nanning 530006, China

**Keywords:** (3-D) coordination framework, 4,4'-oxybis(benzoic acid), self-penetrating topology, fluorescence, electrochemical properties

## Abstract

Assembly of 4,4'-oxybis(benzoic acid) (H_2_L) with manganese chloride in the presence of 2,2'-biphenyl (2,2'-bpy) affords a new coordination polymer [Mn_3_L_3_(2,2'-bpy)_2_]_n_ (**1**), in which the [MnL_2_]_n_ layers are extended by L bridges resulting in a three-dimensional (3-D) coordination framework. The network structure of **1** has unusual (2,6)-connectivity and represents a new type of (8^12^·12^3^)(8)(3) topology. These identical and complementary networks are entangled to generate a self-penetrating supramolecular lattice. Moreover, the fluorescence spectrum of **1** exhibits fluorescent emission in the solution of methanol at room temperature. Electrochemical investigation illustrates the electrochemical properties of the title compound. The structure (C_62_H_40_Mn_3_N_4_O_15_)_n_ is monoclinic with a = 14.2304(18), b = 17.019(2), c = 25.805(3) Å, α = γ = 90, β = 92.932(2)° and space group C2/c.

## 1. Introduction

The design and synthesis of metal-organic frameworks is of great interest due to their intriguing topologic architecture and significant application in many fields [1,2,3,4,5,6,7,8,9]. Among the variety of organic molecules used as linkers in the design of supramolecular networks, heterocyclic N rings and polycarboxylates are the most widely used ligands due to their structural rigidity and coordination mode flexibility [10,11,12,13,14]. In this work we report a novel three-dimensional (3-D) coordination polymer [Mn_3_(L)_3_(2,2'-bpy)_2_]_n_. The network structure of **1** has unusual (2,6)-connectivity and represents a new type of (8^12^·12^3^)(8)(3) self-penetrating topology.

## 2. Results and Discussion

### 2.1. Crystal Structure Descriptions

**Figure 1:**
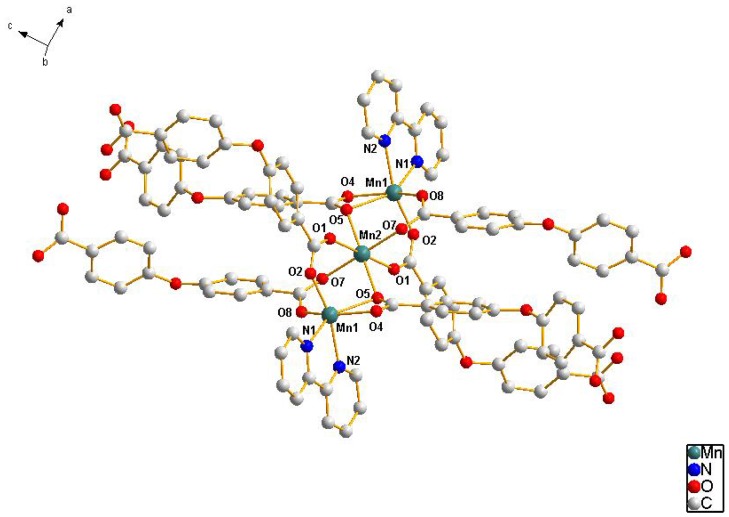
The coordination environment of **1**. All H atoms are omitted for clarity.

The self-assembly of MnCl_2_·4H_2_O with 4,4'-oxybis(benzoic acid) and 2,2'-biphenyl in H_2_O–CH_3_OH mixed solution was performed under weak acid conditions. If NaOH was not added into the reaction system, only unknown ropy precipitate was obtained. If methanol was replaced by ethanol under the same conditions, we could not obtain single crystals. Single crystal X-ray analysis of **1** reveals that the asymmetric unit consists of one and a half Mn(II) ions, one 2,2'-biphenyl and three L ligands, The Mn_1_ coordinate with two nitrogen atoms from one 2,2'-biphenyl (Mn_1_–N 2.261(4)–2.263(4) Å) and four oxygen atoms from three different bridging L ligands (Mn_1_–O 2.074(4)–2.403(4) Å), the Mn_2_ coordinate with six oxygen atoms from six L ligands (Mn_2_–O 2.178(4)–2.245(4) Å), each L ligand connect with four Mn atoms. We can see the environment of Mn in Figure 1. 

**Figure 2:**
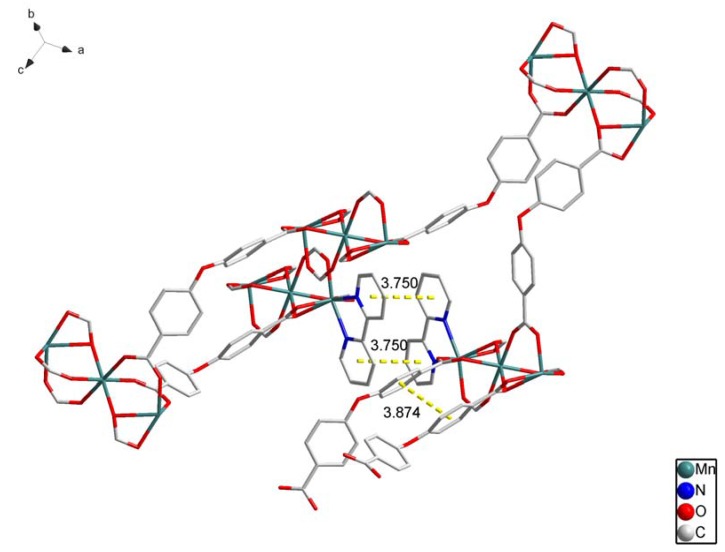
The π-π stacking interactions of **1**. All H atoms are omitted for clarity.

There are many π–π stacking interactions in the crystal (Figure 2). We can see the distance between two benzene rings from diffenent L ligands is 3.875(4) Å, and the dihedral angle is 5.4(4)°. The π–π stacking interactions also exist between one benzene ring and one pyridine ring (3.857(4) Å, 10.68(4)°). 

**Figure 3:**
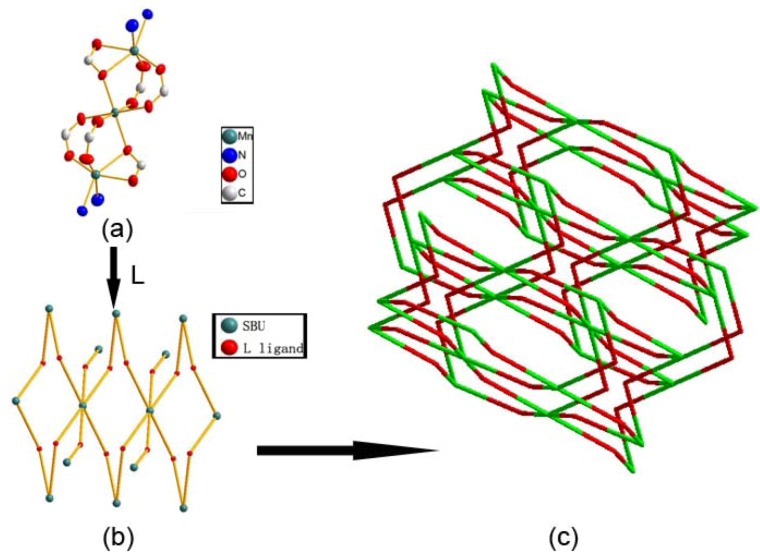
(**a**) Thermal ellipsoid-and-stick representation of SBU; (**b**) Each SBU as a 6-connected node bridged by L ligands to connected with other six SBU nodes; (**c**) A schematic view of the (2,6)-connected (8^12^·12^3^)(8)(3) topology of a 3-D coordination network in **1** (green: SBU; red: L ligand).

**Figure 4:**
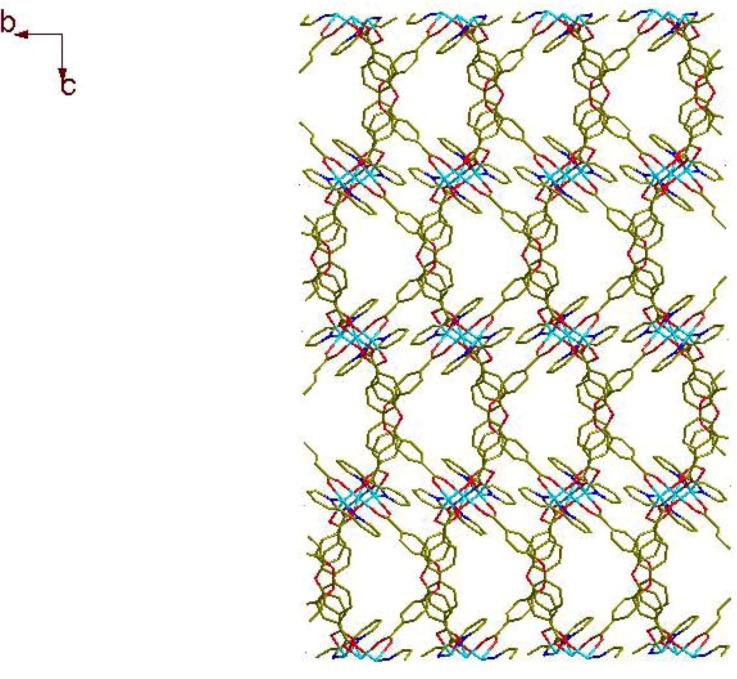
View of the big cavity in the 3-D coordination network.

**Figure 5:**
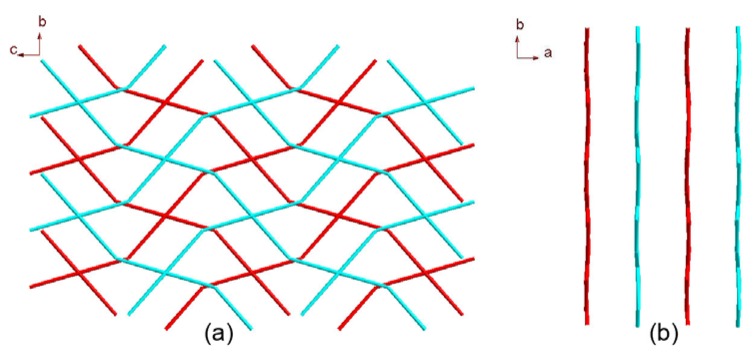
View of the [(SBU)L_4_]_n_ coordination layer along (**a**) the *a* axis and (**b**) the *c* axis.

**Figure 6:**
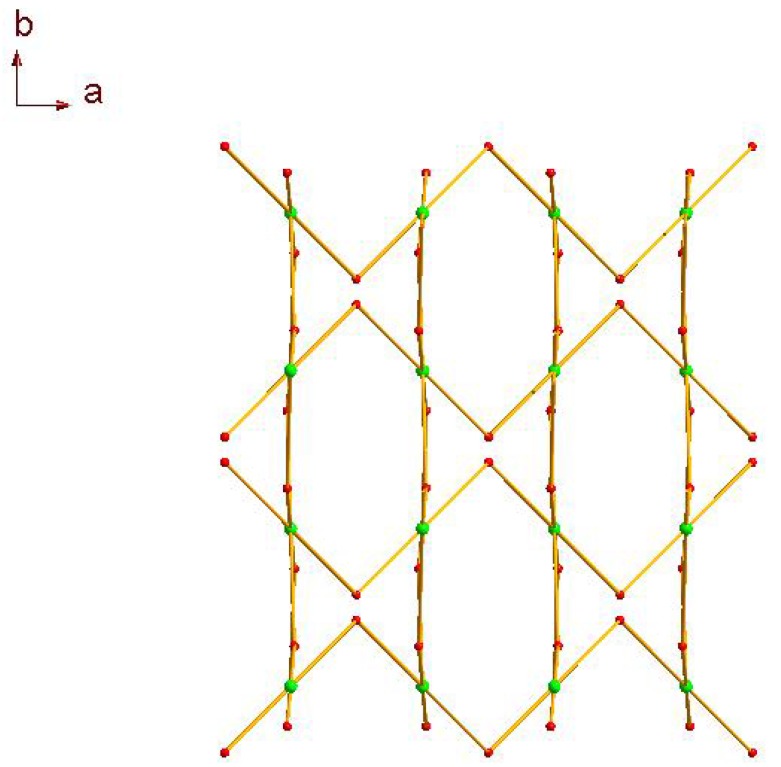
View of the 3-D coordination topology network created by the coordination layer and the L ligand.

**Figure 7:**
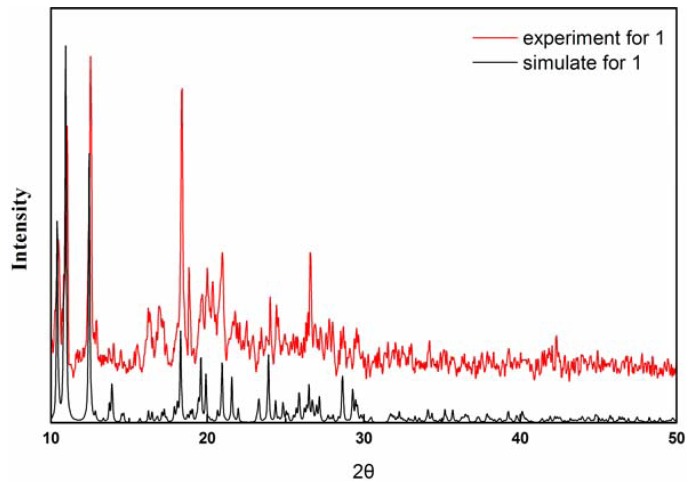
Simulated and experimental XRPD patterns for complex **1**.

The manganese trimetallic unit bridged by six carboxylic groups, constitute a trimetallic second building unit (SBU) [Mn_3_(CO_2_)_6_N_4_] (Figure 3a), with non-bonding Mn·Mn distance of 3.658 Å. The trimetallic SBU, as a 6-connected node Figure 3b, is linked through bridging L to generate an extended 3-D metal-organic framework Figure 3c, and the distances between two cores of SBU are 15.455(3) and 15.049(3) Å, respectively. Long ligands usually lead to large voids and may further result in interpenetration and entangle structures. A large space of 1225.3 Å^3^ (19.6% of the unit-cell volume as evaluated by PLATON [15]) are available to accommodate guest molecule, we can see the big cavity exist in title compound Figure 4. As a result, each core of SBU are connected by four L ligands to generate a coordination layer along the bc plane, as illustrated in Figure 5, in which each SBU center acts as a 4-connected node by linking to four 2-connected ligands, displaying a familiar net topology. The two SBU center binding around each ligand are spaced by the distances of 15.050(3), 15.456(3) Å, respectively. Besides, esch SBU center also linked by the other two L ligands. These two-dimensional (2-D) layers are further extended into a 3-D coordination framework via the other L ligands. Thus, a 3-D (2,6)-connected network with the Schläfli symbol of (8^12^·12^3^)(8)(3) is generated, as depicted in Figure 6. The structural consistency and phase purity of **1** was confirmed by comparing the measured pattern calculated from single-crystal data with the experimental X-ray powder diffraction (XRPD) analysis at room temperature (Figure 7).

### 2.2. Fluorescence Emission Properties

**Figure 8:**
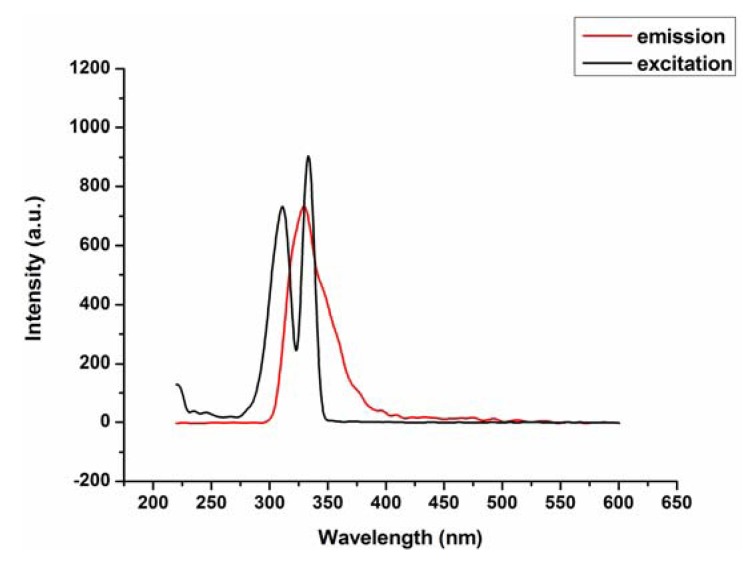
Fluorescence emission spectra of complex **1** in the solution of methanol (λ_ex_ = 312, λ_em_ = 328).

Compound **1** exhibits fluorescence emission around 328 nm upon excitation at 312 nm in the solution of methanol at room temperature (Figure 8), the emission peak of complex 1 is neither metal-to-ligand charge transfer (MLCT) nor ligand-to-metal transfer (LMCT), in nature, since the Mn(II) ions with 3d5 configuration, are difficult to oxidize or reduce, therefore, it may be assigned to the π–π* intraligand fluorescence. 

### 2.3. Electrochemical Properties

**Figure 9:**
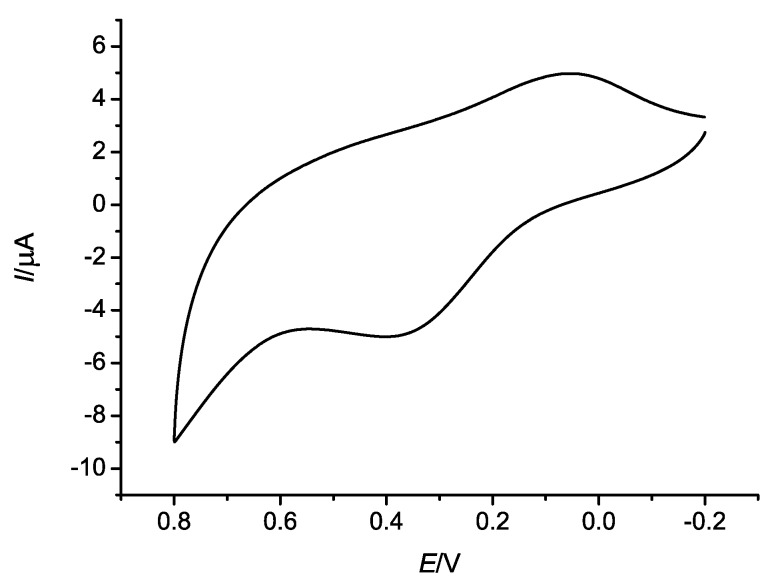
Cyclic voltammograms of complex **1** measured at room temperature.

The electrochemical behavior of complex **1** in methanol and water solution has been investigated by cyclic voltammetry in the potential range from −0.2 to 0.8 V. The resulting cyclic voltammogram (CV) is shown in Figure 9. Complex **1** displays a couple of quasi-reversible oxidation and reduction waves with the reduction potential ranging from −0.1 to 0.2 V and the oxidation potential ranging from 0.2 to 0.5 V. The peaks of the reduction and the oxidation were assigned to the Mn^IV^/Mn^II^ couple which corresponds to one electro-transfer process.

## 3. Experimental

### 3.1. Materials and Instrumentation

All reagents and solvents were used directly as supplied commercially without further purification. Elemental analysis for C, H, and N was carried out on a Perkin-Elmer 2400 II elemental analyzer (Waltham, MA, USA). Excitation and emission spectra were acquired on a Perkin-Elmer instruments LS55 spectrofluorometer (Waltham, MA, USA). The X-ray powder diffraction (XRPD) was recorded on a XD-3 diffractometer (Beijing, China) at 36 kV, 25 mA for a Cu-target tube, and a graphite monochromator. Simulation of the XRPD spectra was carried out by the single-crystal data and diffraction-crystal module of the Mercury (Hg) program available free of charge via the Internet at http://www.iucr.org. Cyclic voltammetry were performed on a CHI760D electrochemical workstation. The three-electrode system consisted of a platinum wire counter electrode, a silver/silver chloride electrode, and a glassy carbon electrode (3.0 mm diameter) as working electrode. All electrochemical experiments were carried out in a conventional electrochemical cell holding methanol and water solution at room temperature.

### 3.2. Synthesis of (3-D) Coordination Framework ***1***

H_2_L (129.1 mg, 0.5 mmol) and 2,2'-biphenyl (77 mg, 0.5 mmol) were dissolved in the mixture of methanol (5 mL) and H_2_O (15 mL). Then an aqueous solution of sodium hydroxide was added dropwise with stirring to adjust the pH value of the solution being 5. Finally, aqueous solution of MnCl_2_·4H_2_O (10 mL, 97 mg, 0.5 mmol) was added. The mixture was kept heated at 130 °C for three days. After cooling to room temperature, the reaction solution was filtered to remove a small quantity of white precipitation. Kept slow evaporation of the filtrate at room temperature and three days later X-ray quality yellow block-shaped single crystals were obtained. The crystals were isolated, washed with carbinol and dried at room temperature (Yield: 65% based on Mn). Calcd. For C_62_H_40_Mn_3_N_4_O_15_: C 59.72, H 3.21, N 4.49%. Found: C 59.70, H 3.22, N 4.48%. 

### 3.3. X-ray Structure Determination

Crystallographic data of the complex was collected on a Bruker SMART CCD diffractometer with graphite monochromated Mo-Ka radiation (λ = 0.71073 Å) at T = 296 K. Absorption corrections were applied by using the multi-scan program [16]. The structure was solved by direct methods and successive Fourier difference syntheses (SHELXS-97), and anisotropic thermal parameters for all nonhydrogen atomswere refined by full-matrix least-squares procedure against *F*^2^ (SHELXL-97) [17]. All nonhydrogen atoms were refined anisotropically. Hydrogen atoms were set in calculated positions and refined by a riding mode, with C–H = 0.93 Å and Uiso(H) = 1.2 Ueq(C) for aromatic H atoms. The potential space of the complex was created by the function of the PLATON. First, we use PLATON to open the cif file. Second, we use the SOLV function, the detail is click the SOLV button, which is just like a water tap, later, we hit RETURN of our keyboard to continue, then we can get the potential space. The crystallographic data and experimental details for the structure analysis are summarized in Table 1, and the selected bond lengths and angles are listed in Table 2. CCDC 869073 contains the supplementary crystallographic data for this paper. These data can be obtained free of charge via www.ccdc.cam.ac.uk/conts/retrieving.html (or from the Cambridge Crystallographic Data Centre, 12, Union Road, Cambridge CB2 1EZ, UK; fax: +44-1223-336033; e-mail: deposit@ccdc.cam.ac.uk).

**Table 1:** Experimental data for complex **1**.

Empirical formula	C_62_H_40_Mn_3_N_4_O_15_
Temperature (K)	1245.80
Wavelength (Å)	296(2)
Crystal system	0.71073
space group	Monoclinic
a (Å)	C2/c
b (Å)	14.230(4)
c (Å)	17.019(2)
α (°)	25.805(3)
β (°)	90
γ (°)	92.932(2)
V (Å^3^)	90
Z	6241.5(9)
Dc (Mg/m^3^)	4
µ (mm^−1^)	1.326
F (000)	0.664
Crystal size (mm)	2540
θ range	0.35 × 0.34 × 0.32
Reflections collected	1.87–25.00
Independent reflections	5493
Completeness to θ = 25.00	5493 [R(int) = 0.0012]
Absorption correction	0.999
Max. and min. transmission	Multi-scan
Data/restraints/parameters	0.8156 and 0.8008
Goodness-of-fit on F	5493/0/381
R indices [I > 2σ(I)]	1.07
R indices (all data)	R1 = 0.0288, wR2 = 0.0732

**Table 2:** The selected bond lenths (Å) and angles (°) for complex **1**.

Mn(1)-O(8) 2.0740(13)	O(1)#3-Mn(2)-O(5)#2 93.51(5)
Mn(1)-O(2)#1 2.0820(13)	O(5)-Mn(2)-O(5)#2 180.0
Mn(1)-O(4) 2.1794(13)	O(4)-Mn(1)-N(2) 90.99(6)
Mn(1)-N(1) 2.2612(15)	N(1)-Mn(1)-N(2) 71.66(6)
Mn(1)-N(2) 2.2625(17)	O(8)-Mn(1)-O(5) 93.82(5)
Mn(1)-O(5) 2.4032(12)	O(2)#1-Mn(1)-O(5) 105.45(5)
Mn(2)-O(7) 2.1782(12)	O(4)-Mn(1)-O(5) 56.89(4)
Mn(2)-O(7)#2 2.1782(12)	N(1)-Mn(1)-O(5) 148.94(5)
Mn(2)-O(1)#1 2.1801(12)	N(2)-Mn(1)-O(5) 91.70(6)
Mn(2)-O(1)#3 2.1801(12)	O(7)-Mn(2)-O(7)#2 180.00(6)
Mn(2)-O(5)#2.2451(12)	O(7)-Mn(2)-O(1)#1 90.51(5)
Mn(2)-O(5)#2 2.2451(12)	O(7)#2-Mn(2)-O(1)#1 89.49(5)
O(1)-Mn(2)#4 2.1801(12)	O(7)-Mn(2)-O(1)#3 89.49(5)
O(2)-Mn(1)#5 2.0821(13)	O(7)#2-Mn(2)-O(1)#3 90.51(5)
O(8)-Mn(1)-O(2)#1 92.42(6)	O(1)#1-Mn(2)-O(1)#3 180.0
O(8)-Mn(1)-O(4) 150.68(5)	O(7)-Mn(2)-O(5) 87.41(5)
O(2)#1-Mn(1)-O(4) 96.77(6)	O(7)#2-Mn(2)-O(5) 92.59(5)
O(8)-Mn(1)-N(1) 111.11(6)	O(1)#1-Mn(2)-O(5) 93.51(5)
O(2)#1-Mn(1)-N(1) 92.13(5)	O(1)#3-Mn(2)-O(5) 86.49(5)
O(4)-Mn(1)-N(1) 96.35(5)	O(7)-Mn(2)-O(5)#2 92.59(5)
O(8)-Mn(1)-N(2) 88.19(6)	O(7)#2-Mn(2)-O(5)#2 87.41(5)
O(2)#1-Mn(1)-N(2) 162.76(6)	O(1)#1-Mn(2)-O(5)#2 86.49(5)

Symmetry codes: #1 x,−y,z+1/2; #2 −x+3/2,−y+1/2,−z+1; #3 −x+3/2,y+1/2,−z+1/2; #4 −x+3/2,y−1/2,−z+1/2; #5 x,−y,z−1/2; #6 −x+2,y,−z+1/2.

## 4. Conclusions

In summary, a novel 3-D Mn(II) coordination polymer was obtained with the aid of H_2_L and 2,2'-biphenyl, which displays a new type of (2,6)-connected (8^12^·12^3^)(8)(3) self-penetrating topological network. Notably, this mode of coordination links three metal ions together and to be a SBU it represents the first appearance in the ligand of H_2_L. Compound **1** exhibits fluorescence in methanol solution at room temperature. We could also clearly observe some electrochemical properties in the title compound.

## Supplementary Materials

Supplementary materials can be accessed at: http://www.mdpi.com/1420-3049/17/9/11103/s1.
